# Molecular Mechanisms for High Hydrostatic Pressure-Induced Wing Mutagenesis in *Drosophila melanogaster*

**DOI:** 10.1038/srep14965

**Published:** 2015-10-08

**Authors:** Hua Wang, Kai Wang, Guanjun Xiao, Junfeng Ma, Bingying Wang, Sile Shen, Xueqi Fu, Guangtian Zou, Bo Zou

**Affiliations:** 1 State Key Laboratory of Superhard Materials, Jilin University, Changchun, 130012, P. R. China; 2 College of Life Science, Jilin University, Changchun, 130012, P. R. China

## Abstract

Although High hydrostatic pressure (HHP) as an important physical and chemical tool has been increasingly applied to research of organism, the response mechanisms of organism to HHP have not been elucidated clearly thus far. To identify mutagenic mechanisms of HHP on organisms, here, we treated *Drosophila melanogaster* (*D. melanogaster*) eggs with HHP. Approximately 75% of the surviving flies showed significant morphological abnormalities from the egg to the adult stages compared with control flies (p < 0.05). Some eggs displayed abnormal chorionic appendages, some larvae were large and red, and some adult flies showed wing abnormalities. Abnormal wing phenotypes of *D. melanogaster* induced by HHP were used to investigate the mutagenic mechanisms of HHP on organism. Thus 285 differentially expressed genes associated with wing mutations were identified using Affymetrix Drosophila Genome Array 2.0 and verified with RT-PCR. We also compared wing development-related central genes in the mutant flies with control flies using DNA sequencing to show two point mutations in the *vestigial* (*vg*) gene. This study revealed the mutagenic mechanisms of HHP-induced mutagenesis in *D. melanogaster* and provided a new model for the study of evolution on organisms.

High hydrostatic pressure(HHP), a physical parameter, which can produce aunique effect on biological systems, has caused great interest of interdisciplinary researchers such as biophysicists and biochemists^1^,^2^,^3^,^4^. During the past decades, the influence of HHP have been investigated in many aspects, including proteins, DNA, enzymes, lipids, viruses, microorganisms, mammalian cells, and tissues^5^,^6^,^7^. With the growing knowledge and understanding of this area, HHP has been applied in different fields such as industry, bioscience and medicine. For example, modulating food functionality, disaggregating proteins, refolding recombinant human interferon, and preparing viral vaccines^8^. Interesting new applications of HHP have also recently emerged. HHP treatment reportedly induces beneficial mutagenesis in microorganisms^9^,^10^ and plants^11^. Therefore, HHP might serve as a useful technique to research the evolution of organisms. However, until now, HHP-induced mutagenesis has not been reported in animals. In addition, the response mechanisms of HHP in organisms are not well understood. Thus, we applied HHP for the first time to induce mutagenesis in *Drosophila melanogaster* (*D. melanogaster*). *D. melanogaster* has been used as a powerful genetic model for approximately one century because many genes and signal pathways are evolutionarily conserved between humans and *D. melanogaster*^12^. The wings of *D. melanogaster* also provide an excellent model for studying the response mechanisms of stress resistance in organisms^13^. Herein, we screened *D. melanogaster* with abnormal wings from mutant populations induced by HHP, and we investigated the mechanism for this mutagenesis using DNA microarray, RT-PCR and DNA sequencing technologies. Our objectives may facilitate identifying and characterizing the mechanisms of HHP mutagenesis in organisms and elucidate the molecular underpinnings for the application of HHP to the evolution of organisms.

## Results

### Screen mutant strains

**Figure 1:**
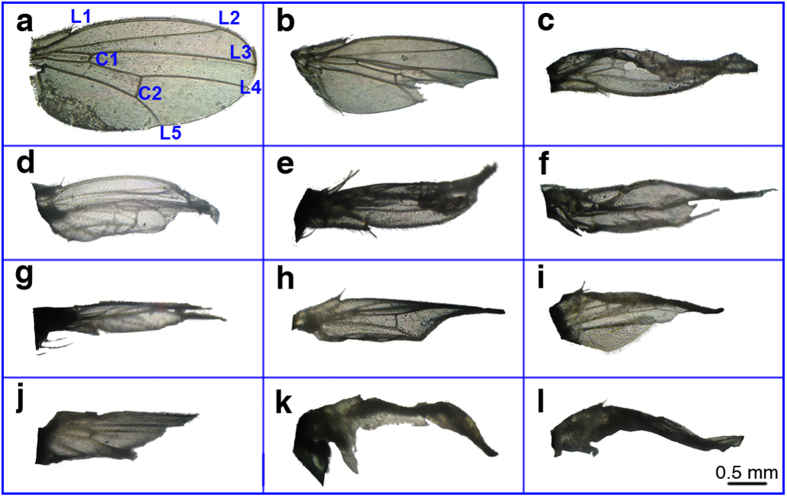
Abnormal wing phenotypes of *Drosophila melanogaster* (*D. melanogaster*) induced by high hydrostatic pressure (HHP). (**a**) The wing shape of *D. melanogaster* in the absence of HHP treatment. Wings in control *D. melanogaster* have five longitudinal veins (L1–L5, representing the first through the fifth longitudinal veins, respectively) and two cross veins (C1 and C2, representing the first and second cross veins, respectively) as well as established wing margins. (**b–l**) Abnormal wings in *D. melanogaster* induced by HHP treatment. The images show the degradation and modifications in wing size, shape, vein patterning, and margin formation.

**Figure 2:**
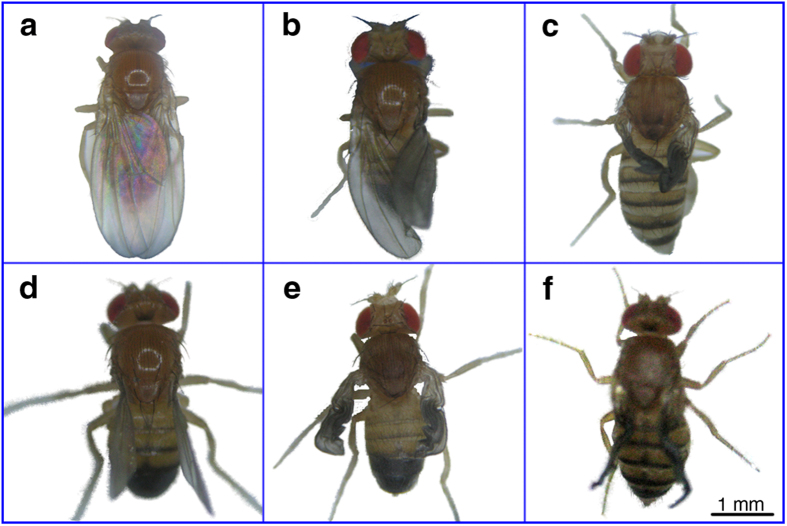
The mutant phenotypes of *D. melanogaster* with HHP induction. (**a**) *D. melanogaster* in the absence of HHP treatment. Normally, the wing is oval shaped, with a length twice that of the abdomen, normal shape and established margin. (**b–f**) HHP-induced mutants show wing degradation and obvious modifications in wing size, shape, and margin formation.

Figure 1 shows the wing morphs induced by HHP in *D. melanogaster*. To determine whether HHP induced mutagenic effects in *D. melanogaster*, we treated *D. melanogaster* eggs using a pressure of 60 MPa for 20 min at room temperature. Approximately 75% of the flies that survived the HHP treatment showed significant morphological abnormalities from the egg stage to the adult stage compared with control flies (*p* < 0.05). The eggs displayed abnormal chorionic appendages, the larvae were large and red, the pupae had melanotic tumors, and the adult flies showed wing abnormalities. Normally, the wing is oval shaped, with a length two times than that of the abdomen and five longitudinal veins and two cross veins as well as established wing margins. However, the abnormal wings of *D. melanogaster* treated with HHP showed degradation and obvious modifications in size, shape, vein patterning, and margin formation (Fig. 1), resulting in an inability to fly. Thus, wing mutant phenotypes were selected for investigating the mechanisms of HHP-induced mutagenesis (Fig. 2).

### Microarrays analysis

**Figure 3:**
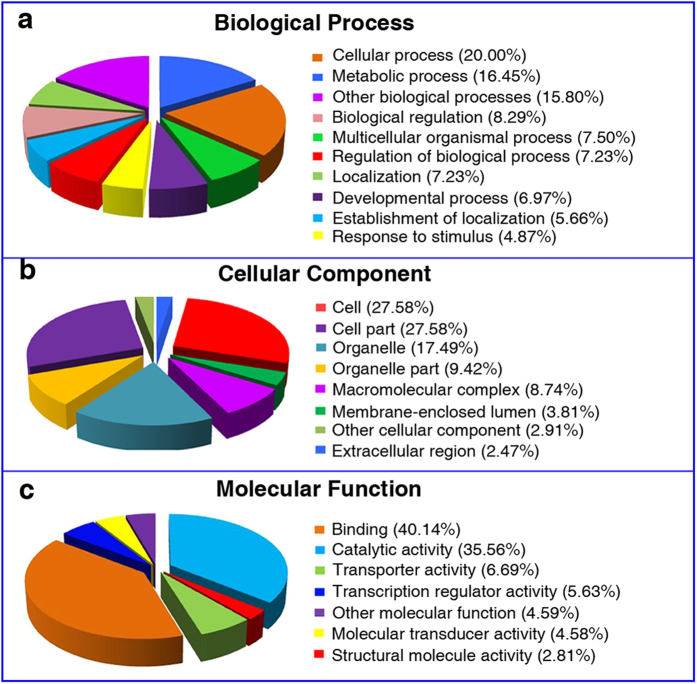
Summary of Gene Ontology (GO) terms associated with HHP-induced wing mutations in *D. melanogaster*. (**a**) The biological process. (**b**) The cellular component. (**c**) The molecular functions.

We hypothesized that differential gene expression produced the HHP-induced wing mutations in *D. melanogaster*. Therefore, we compared patterns of gene expression in mutant flies with those in controls using DNA microarrays. The results revealed that the expression of 285 genes changed at least 2-fold (*P* < 0.05) (Supplementary Table S1). To investigate the biological functions of these differentially expressed genes, we performed a Gene Ontology (GO) category analysis. The differentially expressed genes were assigned to the categories of biological process, cellular component, and molecular function. The biological process GO categories with significant differential representation included metabolic process, cellular process, biological regulation, etc. (Fig. 3a). The cellular component categories enriched in the GO term analysis included cell, cell part, organelle, etc. (Fig. 3b). The molecular functions enriched GO categories included catalytic activity, binding, transporter activity, etc. (Fig. 3c). In addition, our experimental results showed that the HHP treatment altered many signaling pathways in *D. melanogaster*. Among them, the *Notch* signaling pathway plays a direct role in wing development of *D. melanogaster*. *Notch* signaling is mediated by the down-regulated expression of *Numb*, *Tom*, and *sca* and the up-regulated expression of *bun* and *Nedd4* (Supplementary Table S1). To confirm the microarray results, we examined the 10 genes related to wing development using RT-PCR and found significant differences in gene expression. RT-PCR was conducted using primers specific to each of these 10 genes (Supplementary Table S2). Most (2/3 of the less abundant and 7/7 of the more abundant sequences) were verified. Considering that amplification of RNA potentially introduces bias into transcript abundance assays, the results were remarkably concordant (Supplementary Table S3).

### DNA sequencing analysis

**Figure 4:**
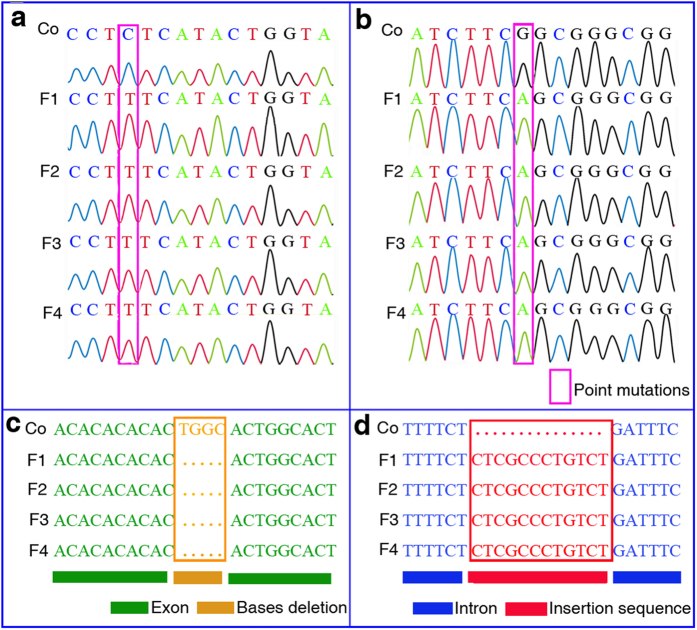
The vestigial (*vg*) gene mutations of *D. melanogaster* induced by HHP treatment. (**a**) The transversion from C to T at nucleotide 1892 in complete cDNAs of *vg* gene. (**b**) The transversion from G to A at nucleotide 2129 in complete cDNAs of *vg* gene. (**c**) The four bases deletion (TGGC) spanning from nucleotide 1739 to 1742 in complete cDNAs of *vg* gene. (**d**) An insertion sequence with 12 nucleotides (5′-CTCGCCCTGTCT -3′) between the second and third exons of *vg*. Co denotes control; F1, F2, F3, and F4 represent first, second, third, and fourth generation mutants, respectively.

To further investigate the mechanisms for the HHP-induced mutagenesis, we performed DNA sequencing, analyzing the *vestigial* (*vg*) and *scalloped* (*sd*) genes, which are involved in wing development. The sequencing results indicated that the *sd* gene sequence remained unaltered; however, two point mutations, four bases deletion and an insertion sequence occurred in the *vg* gene of *D. melanogaster*. The first point mutation was a transversion from C to T at nucleotide 1892 in complete cDNAs of *vg* gene (Fig. 4a) and was a synonymous mutation. The second point mutation located in exon 3, was transversion from G to A at nucleotide 2129 in complete cDNAs of *vg* gene (Fig. 4b) and this mutations was also synonymous mutation. In addition, Our DNA sequencing results exhibited four bases deletion (TGGC) spanning from nucleotide 1739 to 1742 in complete cDNAs of *vg* gene (Fig. 4c), and an insertion sequence with 12 nucleotides (5′-CTCGCCCTGTCT-3′) between the second and third exon of *vg* (Fig. 4d). All mutations were stably inherited for four generations.

## Discussion

**Figure 5:**
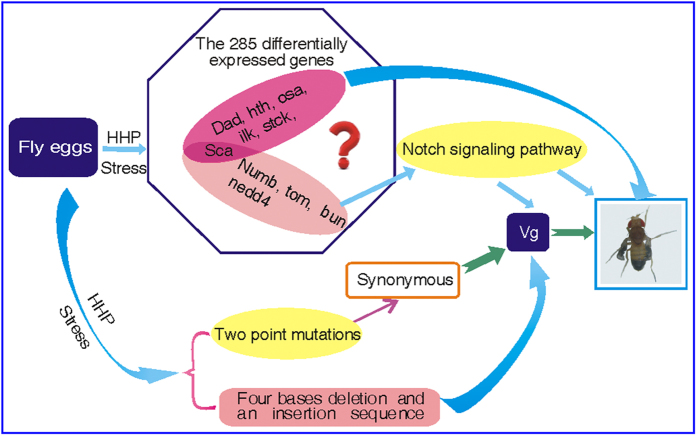
Model of the molecular mechanism for the wing mutagenesis induced by HHP treatment in *D. melanogaster*. HHP stress induces 285 differentially expressed genes in the mutants. Among them, 6 genes, including *sca*, *hth*, *osa*, *dad, ilk,* and *stck* have a direct relationship with wing development. In addition, *Numb*, *tom*, *sca*, *bun*, and *nedd4* genes have an indirectly role in *D. melanogaster* wing development through *Notch* signaling pathways. The *sca* gene is common to both. The *Notch* signaling pathway also affects *D. melanogaster* wing phenotype via the *vg* target gene, and HHP stress induces two point mutations in the *vg* gene. The two mutations are synonymous mutations. Furthermore, the four bases deletion in the exon 1 of *vg* gene resulting in the change of reading frame and the consequent failure to generate functional protein. An insertion sequence between the second and third exon of *vg* indirectly affect the function of protein. The coordinated interaction of these abnormal genes is crucial for the wing mutagenesis induced by HHP treatment in *D. melanogaster*.

*D. melanogaster* is a typical model organism for studying the response mechanisms of stress resistance. The present study took advantage of this model, by utilizing three molecular strategies, DNA microarray, RT-PCR and DNA sequencing, to identify potential causative genes underlying HHP-induced mutagenesis. With regard to the effect of HHP on organisms, previous experiments were performed on microorganisms and plants^11^. For example, Yayanos *et al.* reported that the synthesis of DNA, RNA, and protein was inhibited by HHP in living *E. coli* cells. Zhang *et al.* indicated that HHP impaired DNA strand integrity in rice. However, their results just limited the effect of HHP on biopolymer such as DNA, RNA, and protein and did not determine specific genes pertaining to HHP mutagenesis. By contrast, our experiments, using *D. melanogaster* as an animal model, provided further evidence for the mechanisms of the HHP-induced mutations in terms of the analysis of gene differential expression and DNA sequence variation. Specifically, we identified 285 differentially expressed genes in the mutants. We assigned these genes to the subcategories of molecular function, cellular component, and biological process. The 10 genes related to wing development were screened and verified using RT-PCR, with a remarkable concordance in the results. Among them, 6 genes, including *sca*, *hth*, *osa*, *dad*, *Ilk*, and *stck*, played important roles in wing development and directly induced the wing mutations in *D. melanogaster*. Cell signaling pathways regulate important physiological processes, such as cell proliferation, differentiation, growth, and development using various mechanisms^14^,^15^,^16^. Our experimental results implicate multiple signaling pathways in the acquisition of HHP tolerance in *D. melanogaster* mutants. Among them, the *Notch* signaling pathway plays important roles in the wing development of *D. melanogaster*. *Notch* signaling is mediated by the down-regulated expression of *Numb*, *Tom*, and *sca*, and the up-regulated expression of *bun* and *Nedd4* (Supplementary Table S1 and Supplementary Table S3). In general, the normal wing development in *D. melanogaster* requires the coordinated actions of several genes, such as *vg*, *sd*, *Notch*, and *wg*^17^. The *vg* gene in *D. melanogaster* not only plays a central role in wing development but is also a target of the *Notch* signaling pathway^18^,^19^. In addition, *vg* expression occurs throughout the entire developing wing field^20^. Loss of *vg* results in wing development failures, and ectopic expression of *vg* leads to the development of ectopic wings^21^. For *D. melanogaster*, the *vg* gene is located on chromosome 2, and the complete sequence contains seven exons and eight introns. Our DNA sequencing results indicated that two point mutations occurred in exon 3 of *vg* gene, they were synonymous mutations. Synonymous mutations indirectly affect the function of proteins^22^,^23^,^24^. Furthermore, the four bases deletion in the exon 1 of *vg* gene was done in *D. melanogaster* mutant, resulting in the change of reading frame and the consequent failure to generate functional protein. Generally, frameshift mutations are base deletions or additions within the coding region of a gene. As expected, because of potential problems with disturbing the reading frame, the entire set of triplets downstream of the deletion or addition is altered. In many cases, the deletion or addition results in the presence of in-frame termination sequences which stop the product. Frameshift mutations may therefore result in more severe phenotypic effects than do a number of the base changes which cause either silent or conservative mutations in protein products^25^. In addition, our DNA sequencing results showed an insertion sequence with 12 nucleotides (5′-CTCGCCCTGTCT-3′) between the second and third exons, namely, the junction of exon 3 and the intron 3 of *vg* gene. This insertion sequence was another incentive for abnormal wing development of *D. melanogaster* induced by HHP. The coordinated interaction of these ectopic expression genes and *vg* gene mutants is crucial for abnormal wing development in *D. melanogaster* induced by HHP treatment. However, how these genes induced by HHP regulate abnormal wing development of *D. melanogaster* is not well understood and needs further study. Our results suggest the model shown in Fig. 5 for the molecular mechanism of wing mutagenesis induced by HHP treatment in *D. melanogaster*.

To summarize, HHP-induced morphological mutagenesis in *D. melanogaster* was presented systematically. Through DNA microarray, RT-PCR and DNA sequencing, three molecular strategies were used to investigate the molecular mechanism of wing mutagenesis in *D. melanogaster*, examining 285 differentially expressed genes and *vg* gene mutations. Our findings provided new evidence for the mechanisms of HHP mutagenesis in organisms and indicated the need for additional elucidation. Furthermore, our results suggested the molecular underpinnings of HHP-induced alterations in the evolution of organisms.

## Methods

### Origin and maintenance of experimental flies

*Drosophila melanogaster* (*D. melanogaster*) used in this study originated from the Institute of Genetics and Cytology, Northeast Normal University, Changchun (China). The flies were maintained according to the report by Sørensen’s. *et al.*^26^.

### High hydrostatic pressure (HHP) treatment

For HHP experiments, *D. melanogaster* eggs were collected from healthy flies by placing a fresh food large plate in the big box for 1 h. These eggs were transferred into sterile bags (300 eggs/bag). Subsequently, each bag with eggs was exposed to pressure of 60 MPa for 20 min at room temperature. The control eggs were maintained under normal atmospheric pressure (0.1 MPa).

### Screen mutant strains

To screen mutant strains, the flies that survived the HHP treatment were observed with electron microscope and stereomicroscope, and screened different mutants at all stages from eggs to adult flies.

### Gene expression analysis using microarrays

DNA microarray analyses were performed according to the protocols of Affymetrix and the methods described previously^27^,^28^.

### Data analysis and Statistical analysis

Data analysis and statistical analyses were performed using the SBC Analysis System from the website http://sas.ebioservice.com/.

### RT-PCR analyses

Total RNA was isolated from the control and mutant flies using RNAiso Plus (Takara, China). RT-PCR analyses were performed according to previously published protocols^29^,^30^. The sequences of primers for RT-PCR listed in Supplementary Table S2.

### DNA sequencing

Genomic DNA was extracted from individual fly using a unique method in its own right. Polymerase chain reaction (PCR) was performed in a volume of 22 uL that contained 1 μL of template DNA (100 ng/μL), 0.4 μL of DNA polymerase (Taq DNA polymerase, 5 U/μL), 2.0 μL of 10 × buffer, 1 μL of dNTPs (2.5 mM each), and 0.4 μL of each primer (10 μM), and 16.8 μL sterilized double distilled water. The conditions for PCR were as follows: denaturation for 5 min at 94 °C followed by 45 sec at 94 °C, annealing for 30 sec at 59 °C, and extension for 1 min at 72 °C. This procedure was repeated for 35 cycles with a final 7 min extension at 72 °C. The amplified products were separated on 2% agarose gels. The PCR products were excised from the gel, purified with the AxyPrep DNA gel extraction kit, and sequenced.

## Additional Information

**How to cite this article**: Wang, H. *et al.* Molecular Mechanisms for High Hydrostatic Pressure-Induced Wing Mutagenesis in Drosophila melanogaster. *Sci. Rep.* **5**, 14965; doi: 10.1038/srep14965 (2015).

## Supplementary Material

Supporting Information
