# Supplementary material for: Molecular Mechanisms for High Hydrostatic Pressure-Induced Wing Mutagenesis in Drosophila melanogaster
Source: Sci Rep. 2015 Oct 8;5:14965. doi: 10.1038/srep14965 (PMC4597337; doi:10.1038/srep14965)
Supplement: Supporting Information [file srep14965-s1.pdf]

Supplementary Information

Molecular Mechanisms for High Hydrostatic Pressure-Induced Wing Mutagenesis in *Drosophila melanogaster*

Hua Wang[1, 2, +], Kai Wang[1, +], Guanjun Xiao[1, +], Junfeng Ma[2], Bingying Wang[2], Sile Shen[2], Xueqi Fu[2,*], Guangtian Zou[1], and Bo Zou[1,*]

1. State Key Laboratory of Superhard Materials, Jilin University, Changchun, 130012, P. R. China

2. College of Life Science, Jilin University, Changchun, 130012, P. R. China

* E-mail: zoubo@jlu.edu.cn. Phone: (86) 431-85168883. Fax: (86) 431-85168881.

* E-mail: fxq@jlu.edu.cn. Phone: (86) 431-85155152. Fax: (86) 431-85155152.

+ These authors contributed equally to this work

Supplementary Table S1. The 285 differentially expressed genes of fly with wing abnormalities identified using DNA microarray

Probe_Set_ID	Gene_Symbol	Alignments	FCAbsolute(mutation vs_control)	regulation	p_value
1623892_at	*Bsg25A*	chr2L:4683049-4684459 (-) // 100.0 //	38.1700	down	0.0130
1624476_at	*Tom*	chr3L:14962446-14963417 (+) // 100.0 //	16.9039	down	0.0070
1632572_at	*CG34214*	chr2R:20552973-20553479 (-) // 100.0 //	28.1312	down	0.0061
1627407_at	*SNCF*	chr3L:13371684-13372295 (+) // 99.35 //	13.5565	down	0.0006
1638461_s_at	*CG7271 /// term*	chr3L:18581862-18583243 (+) // 100.0 // /// chr3L:18585423-18586865 (-) // 98.99 //	21.8353	down	0.0164
1636998_at	*sca*	chr2R:8668048-8689515 (+) // 100.0 //	1.6978	down	0.0339
1635277_at		chr3L:23753367-23758504 (-) // 95.33 //	1.8489	down	0.0499
1624702_s_at	*dpr10*	chr3L:10140552-10179205 (-) // 100.0 //	1.6663	down	0.0154
1626255_at	*Gr61a*	chr3L:841717-843411 (+) // 100.0 //	1.5499	down	0.0055
1631378_at	*beat-Ia*	chr2L:16049994-16081688 (+) // 100.0 //	1.8730	down	0.0363
1628865_at	*opa*	chr3R:678566-695705 (+) // 99.86 //	1.8663	down	0.0318

1623217_at	*CG8743*	chr3L:19738375-19742866 (-) // 100.0 // /// chr3L:19706959-19711450 (-) // 100.0 //	1.5214	up	0.0290
1628702_at	*Muc14A*	chrX:15903160-15955708 (+) // 67.32 //	1.6948	up	0.0265
1623104_at	*Smc5*	chr3L:21562376-21566624 (+) // 100.0 //	1.5068	down	0.0187
1629154_at	*Or43a*	chr2R:3120343-3122418 (-) // 100.0 //	1.6179	down	0.0158
1634323_at	*CG17191*	chr3R:22844329-22845473 (-) // 100.0 //	3.0227	down	0.0147
1629828_at	*CG13375*	chrX:180615-184374 (-) // 100.0 //	2.1938	down	0.0112
1638123_at		chr3R:11830341-11833795 (-) // 100.0 //	1.3771	up	0.0042
1631171_at	*CheB93a*	chr3R:17233242-17233905 (+) // 100.0 //	3.2950	up	0.0410
1631583_at		chr3L:7459569-7461362 (+) // 100.0 //	1.5894	up	0.0317
1623696_at	*CG15634*	chr2L:4687512-4688956 (+) // 100.0 //	2.5058	down	0.0288
1624842_at	*koi*	chr2R:2702564-2704510 (+) // 100.0 //	1.8156	up	0.0287
1633058_at	*halo*	chr2L:1517531-1518184 (-) // 100.0 //	9.4982	down	0.0269
1638878_at	*CG17302 /// Or23a*	chr2L:2655393-2660830 (+) // 100.0 //	1.4675	down	0.0160

1639039_at	*Csk*	chr3R:7467992-7468796 (-) // 100.0 //	1.7196	down	0.0159
1629003_at	*CG6208*	chr3R:5946244-5947096 (-) // 100.0 //	1.4989	down	0.0436
1632625_a_at	*CG8960*	chr3L:2197012-2198107 (+) // 100.0 //	4.2868	down	0.0208
1639684_at		chr3R:17666626-17667632 (+) // 100.0 //	1.5623	up	0.0136
1636820_s_at	*spo*	chr3L:5583182-5585677 (-) // 100.0 //	2.6636	down	0.0010
1628762_at		chr2L:12851880-12853181 (+) // 100.0 //	1.4772	up	0.0308
1627159_at	*CG13427*	chr2R:16426452-16426770 (-) // 100.0 //	10.7211	down	0.0369
1624225_at	*danr*	chr3R:20963798-20965625 (+) // 100.0 //	1.3005	down	0.0248
1627158_at		chr3L:18026596-18027582 (+) // 100.0 //	1.8660	up	0.0458
1638656_at	*CG8646*	chr2R:8543460-8546853 (+) // 100.0 //	1.6165	down	0.0269
1640076_at	*CG31960*	chr2L:3784945-3785515 (+) // 100.0 //	1.5250	up	0.0310
1629540_a_at	*bw*	chr2R:19415327-19426016 (-) // 100.0 //	1.3278	down	0.0351
1629175_at		chr2L:12414959-12415139 (-) // 100.0 //	1.7805	up	0.0461
1626672_at	*CG1399*	chr2R:3575700-3582699 (-) // 98.55 //	1.2621	down	0.0459

1629421_at	*CG16789*	chr3R:5391953-5394918 (+) // 100.0 //	1.6794	up	0.0465
1623532_at	*CG34236*	chr2R:9491917-9492839 (-) // 100.0 //	1.3859	down	0.0441
1638484_at	*Hsp67Bc*	chr3L:9364817-9365417 (-) // 100.0 //	1.3659	up	0.0475
1627611_at	*CG42269*	chr3L:6059594-6059993 (+) // 100.0 //	1.2955	down	0.0440
1625400_at	*Cpr47Ea*	chr2R:7139631-7140396 (-) // 100.0 //	3.0537	down	0.0466
1632376_at	*CG5644*	chr3L:8970494-8972980 (-) // 100.0 //	1.6027	down	0.0104
1631886_at		chr3L:11685199-11686434 (+) // 100.0 //	1.5921	down	0.0392
1638710_at	*CG14367*	chr3R:9519774-9521484 (+) // 100.0 //	1.4516	up	0.0015
1636132_at		chr3R:16103018-16104885 (+) // 100.0 //	1.2112	up	0.0192
1636952_at		chr2R:17922425-17923454 (+) // 100.0 //	1.4117	up	0.0117
1637153_at	*Dis3*	chr3R:20159144-20162971 (+) // 100.0 //	1.3454	down	0.0049
1624745_at	*Ilp5*	chr3L:9816575-9816973 (-) // 100.0 //	1.6240	down	0.0067
1640631_at	*CG32809*	chrX:1569112-1586580 (-) // 100.0 //	1.3439	down	0.0049
1637791_at	*CG31922*	chr2L:1170233-1170847 (+) // 100.0 //	1.4817	down	0.0151

1632337_at	*Sur*	chr2L:10182556-10198641 (-) // 100.0 //	1.3270	down	0.0076
1633908_at	*Pdf*	chr3R:22281181-22281863 (-) // 100.0 //	1.4571	up	0.0086
1623846_at	*disco-r*	chrX:16012335-16017227 (-) // 100.0 //	1.8766	up	0.0204
1624212_at	*CG6784*	chr3R:19198898-19199766 (+) // 100.0 //	1.6603	up	0.0443
1625036_at	*CG18281*	chr3L:20492935-20494600 (+) // 100.0 //	1.4845	down	0.0328
1639829_at	*CG15382*	chr2L:2155759-2156791 (+) // 100.0 //	2.1062	down	0.0181
1633780_at	*CG13793*	chr2L:7717051-7719056 (-) // 100.0 //	1.5639	down	0.0491
1634620_a_at	*stl*	chr2R:18698896-18703508 (+) // 100.0 //	1.1564	down	0.0415
1623775_at	*CG31089*	chr3R:21728418-21729812 (+) // 100.0 //	2.1741	up	0.0202
1637859_at	*ATbp*	chrXHet:84921-87149 (+) // 100.0 //	1.2187	down	0.0432
1631524_a_at	*hth*	chr3R:6335561-6439973 (-) // 100.0 //	1.5360	up	0.0055
1640757_at	*CG33462*	chr2R:11582205-11585419 (+) // 100.0 //	4.7204	up	0.0076
1630919_at	*pip*	chr3L:19291629-19330699 (-) // 100.0 //	1.6755	down	0.0478
1632570_at	*CG2052*	chr4:396259-412143 (-) // 100.0 //	1.5671	down	0.0457

1623289_at	*Obp83ef*	chr3R:1935500-1936730 (-) // 100.0 //	1.8101	up	0.0255
1633888_s_at	*kirre*	chrX:2994110-3026824 (+) // 99.66 //	1.2196	up	0.0464
1624721_at	*vimar*	chr2R:2862786-2873214 (-) // 100.0 //	1.2404	down	0.0254
1637713_at	*PH4alphaNE3*	chr3R:26429902-2643186 6 (+) // 100.0 //	1.2749	up	0.0028
1628076_at	*Lip3*	chr3R:9196032-9197474 (-) // 100.0 //	7.1755	up	0.0302
1640149_at	*CG1463*	chrX:12503589-12505632 (-) // 100.0 //	1.4224	down	0.0345
1634177_a_at	*CG31055*	chr3R:23511351-2351385 6 (-) // 100.0 //	1.2979	up	0.0271
1629700_at		chrX:13878760-13879941 (-) // 100.0 //	1.3068	up	0.0468
1628317_at	*CG14410*	chrX:14731006-14731363 (+) // 100.0 //	1.3000	up	0.0264
1634482_at	*CG8918*	chrX:17094758-17096631 (-) // 99.76 //	1.7205	up	0.0498
1625940_s_at	*sim*	chr3R:8883480-8903950 (+) // 100.0 //	1.5780	down	0.0038
1632637_at	*hiw*	chrX:14904720-14957440 (-) // 99.97 //	1.2967	down	0.0014
1627632_at	*CG17777*	chrX:2081210-2081501 (+) // 100.0 //	2.0702	down	0.0044
1639200_at	*CG14333*	chr3R:13210373-1321068 2 (+) // 100.0 //	1.3710	up	0.0486

1635592_at	*vav*	chrX:19155863-19167312 (+) // 100.0 //	1.2364	up	0.0435
1631175_a_at	*CG10283*	chr2L:18472952-18484511 (-) // 100.0 //	1.2336	down	0.0456
1629215_at	*Kua*	chr2L:20100946-20102516 (+) // 100.0 //	1.4583	up	0.0051
1628219_at	*CG32050*	chr3L:9818798-9821003 (+) // 100.0 //	1.2838	up	0.0318
1636615_at		chrX:411256-417253 (-) // 100.0 //	1.3491	down	0.0353
1632912_s_at	*CG13667*	chr3L:8398531-8400455 (+) // 100.0 //	1.0942	down	0.0274
1631157_at	*CG7365*	chr3L:20117395-20120465 (-) // 100.0 //	1.3069	down	0.0459
1635940_at	*CG5367*	chr2L:10354809-10356276 (-) // 100.0 //	1.8506	down	0.0092
1627360_at	*pyd*	chr3R:4658908-4672009 (-) // 100.0 //	1.5423	up	0.0397
1626435_at	*CG15263*	chr2L:15285026-15285905 (+) // 100.0 //	3.5060	up	0.0427
1625565_at	*Nmnat*	chr3R:20770958-20772555 (+) // 100.0 //	1.1690	up	0.0363
1629553_at	*CG14153*	chr3L:10636632-10637715 (+) // 100.0 //	1.5476	down	0.0428
1627678_at	*CG18302*	chr2L:10534972-10536315 (+) // 100.0 //	1.2987	down	0.0318
1629741_at	*CG32666 /// DsimCG32666*	chrX:11387202-11449079 (+) // 100.0 //	1.3100	up	0.0186

1629924_at	*CG10238*	chr3R:20951353-2095306<br>8 (-) // 100.0 //	1.2054	down	0.0359
1633572_at		chr2R:7182903-7183317<br>(-) // 100.0 //	2.1026	down	0.0006
1631807_at	*CG9386*	chr3R:5379410-5381102<br>(-) // 100.0 //	1.1324	down	0.0163
1626377_at	*CG8358*	chr3R:5501244-5503501<br>(+) // 100.0 //	1.6966	up	0.0059
1639977_at	*CG16941*	chr3R:12824815-1282807<br>7 (-) // 100.0 //	1.2948	up	0.0303
1624362_at	*Nplp4*	chr2L:2008529-2008855<br>(+) // 100.0 //	1.8253	down	0.0028
1631564_at	*Usp7*	chrX:11916860-11923061<br>(-) // 100.0 //	1.1862	down	0.0420
1628977_at	*RYBP*	chr2R:18558256-1856071<br>7 (+) // 100.0 //	1.2450	down	0.0122
1626819_s_at	*CG11552*	chr3L:15034732-1503567<br>0 (-) // 100.0 //	1.5016	up	0.0074
1639045_at	*mthl5*	chr3R:7709659-7712887<br>(-) // 100.0 //	1.1743	up	0.0402
1627723_at	*CG14701*	chr3R:7057738-7058140<br>(+) // 100.0 //	1.2987	down	0.0353
1636607_at	*Rab2*	chr2R:2584226-2587441<br>(+) // 100.0 //	1.4700	up	0.0312
1629367_at	*CG15534*	chr3R:26257785-2626069<br>8 (+) // 100.0 //	2.4348	up	0.0222
1635937_at	*CG4500*	chr2L:14027009-1402936<br>8 (+) // 99.82 //	2.3251	up	0.0302

1625165_at	*CG17187*	chr3R:7056338-7057578 (-) // 100.0 //	1.1225	up	0.0435
1628572_s_at	*stops*	chr3R:26983876-2699482 5 (-) // 100.0 //	1.5141	up	0.0055
1631435_at	*CG7988*	chr3R:14046247-1404717 3 (+) // 100.0 //	1.1553	down	0.0219
1636524_at	*CG34316 /// mRpS10*	chr3R:11056339-1105733 8 (+) // 100.0 //	2.4320	up	0.0018
1625227_at	*CG30456*	chr2R:12990445-1299504 7 (+) // 99.79 //	1.2859	down	0.0077
1627180_at	*Cyp4d14*	chrX:2067529-2069346 (-) // 100.0 //	1.5293	down	0.0287
1630198_at	*Aly*	chr3R:2905385-2906126 (-) // 100.0 //	1.2083	up	0.0323
1627500_at	*CG30345*	chr2R:5036331-5038809 (+) // 100.0 //	1.1575	down	0.0333
1635196_at	*CG6836*	chr3L:18943731-1894850 9 (+) // 100.0 //	1.2483	down	0.0475
1641267_at	*r*	chrX:16549411-16563110 (+) // 100.0 //	1.2463	down	0.0251
1633792_a_at	*osa*	chr3R:13513539-1354405 9 (-) // 100.0 //	1.1742	up	0.0440
1626116_at	*Sp212*	chr3R:10047849-1004985 6 (-) // 100.0 //	1.6113	up	0.0234
1637582_a_at	*Nedd4*	chr3L:17523182-1753976 0 (+) // 100.0 //	1.2083	up	0.0490
1627204_at	*CG4749*	chr2L:1147832-1149449 (+) // 99.69 //	1.2223	down	0.0492

1638634_at	*CG32448*	chr3L:21820945-21821678 (-) // 100.0 //	1.1455	down	0.0113
1630528_at	*CG17751*	chr3R:15433361-15434804 (+) // 100.0 //	1.6788	down	0.0471
1640257_at	*clt*	chr2R:17504560-17506564 (-) // 100.0 //	1.1389	down	0.0409
1632989_a_at	*mth*	chr3L:343375-346909 (-) // 100.0 //	1.2076	down	0.0465
1623549_at	*CG1973*	chr3R:25574947-25579329 (+) // 100.0 //	1.1277	down	0.0134
1633333_a_at	*CG7231*	chr2L:7983918-7986780 (+) // 100.0 //	1.2502	down	0.0095
1631763_at	*CG31793*	chr2L:19003409-19008231 (+) // 100.0 //	1.1938	up	0.0037
1635020_s_at	*phr6-4*	chr2L:20643347-20645789 (-) // 100.0 //	1.4218	down	0.0047
1627463_at	*Damm*	chr2R:7752699-7753888 (-) // 100.0 //	2.2573	down	0.0291
1635330_at	*CG5428*	chr2R:19569146-19570475 (+) // 100.0 //	1.5075	up	0.0177
1625943_at	*RpS30*	chr3R:16676270-16677156 (+) // 100.0 //	1.3937	down	0.0082
1634798_at	*Rpp30*	chr2L:251162-252331 (-) // 100.0 //	1.2663	down	0.0296
1640386_at	*wbl*	chr2R:15137265-15138419 (+) // 100.0 //	1.3835	down	0.0010
1625844_s_at	*apt*	chr2R:19452419-19487223 (+) // 100.0 //	1.1558	up	0.0472

1637875_a_at	*CG33649 /// DNApol-gamma35*	chr2L:13831479-13833167 (-) // 100.0 //	1.2231	down	0.0498
1629141_at	*InR*	chr3R:17398220-17406251 (-) // 100.0 //	1.2185	up	0.0269
1629040_at	*CG3476*	chr2L:7027593-7028959 (+) // 100.0 //	1.2176	down	0.0494
1628763_at	*Ptpmeg*	chr3L:328120-356050 (+) // 100.0 //	1.2132	down	0.0238
1625075_at	*Nep2*	chr3R:545518-557595 (-) // 100.0 //	1.4571	down	0.0153
1636759_at	*CG8303*	chr2R:12482532-12491739 (-) // 100.0 //	1.9463	down	0.0333
1641063_s_at	*Oatp30B*	chr2L:9521214-9540045 (+) // 100.0 //	1.2839	down	0.0058
1636673_s_at	*ttk*	chr3R:27539605-27558117 (+) // 100.0 //	1.1812	down	0.0204
1632652_s_at	*CG30022 /// CG40100*	chr2RHet:1142179-1142507 (+) // 100.0 // /// chr2R:7274876-7275564 (-) // 90.51 //	1.2597	up	0.0173
1635572_at	*CG30058 /// CG34312*	chr2R:9097859-9098882 (-) // 100.0 //	1.6054	up	0.0314
1635393_s_at	*Eip75B*	chr3L:17946097-17946705 (-) // 100.0 //	1.3723	up	0.0269
1630212_at	*CG2065*	chr2R:3551290-3552774 (+) // 99.5 //	2.0418	down	0.0125
1628990_at	*Hmgcr*	chr3R:19559045-1957686	1.1056	up	0.0244

		8 (+) // 100.0 //			
1625624_at	*CG14105*	chr3L:13419061-13419736 (-) // 100.0 //	1.6656	up	0.0460
1639600_at	*CG13919*	chr3L:1645541-1645937 (+) // 100.0 //	1.0928	down	0.0149
1637825_at	*CG11127*	chr2R:3350875-3352492 (+) // 100.0 //	1.1595	down	0.0203
1623686_at	*CG7148*	chr3L:21823643-21825305 (-) // 100.0 //	1.0838	down	0.0325
1639522_at	*CG31950*	chr2L:2878592-2878958 (+) // 100.0 //	1.1113	down	0.0463
1630530_at	*PrBP*	chr2L:8684814-8685954 (-) // 100.0 //	1.2489	down	0.0258
1626613_at	*CG11236 /// DsimCG11236*	chr2L:6803032-6804372 (+) // 100.0 //	1.5597	up	0.0287
1640249_at	*CG33173*	chrX:15667203-15674095 (+) // 100.0 //	1.8865	down	0.0360
1627020_at	*CG11110*	chr2R:16529306-16529922 (+) // 100.0 //	1.3376	down	0.0425
1637774_s_at	*CG8389*	chr2R:11895876-11901329 (+) // 100.0 //	1.1664	down	0.0452
1632180_at	*CG7791*	chr2R:1645559-1647896 (-) // 100.0 //	1.1003	down	0.0173
1634804_at		chr2L:7472504-7474893 (-) // 100.0 //	1.0974	up	0.0476
1630814_s_at	*CG10353*	chrX:11771768-11781192 (-) // 100.0 //	1.1735	up	0.0039

1625387_s_at	*E2f /// torp4a*	chr3R:17446025-17486127 (-) // 100.0 //	1.0612	down	0.0480
1637768_at	*CG30100 /// CG42372*	chr2R:12178957-12179760 (+) // 100.0 //	1.2939	down	0.0143
1641304_s_at	*CG9801*	chr3R:4561600-4573372 (-) // 100.0 //	1.1606	up	0.0194
1624478_at	*Roc1a*	chrX:541319-542636 (+) // 100.0 //	1.1454	down	0.0363
1633556_s_at	*Dad*	chr3R:12879728-12896135 (+) // 100.0 //	1.3437	up	0.0251
1638158_at	*psidin*	chr3R:15847424-15852207 (+) // 100.0 //	1.1243	down	0.0327
1639064_s_at	*Akt1*	chr3R:11924937-11930239 (+) // 100.0 //	1.4977	up	0.0141
1637056_s_at	*CG11200*	chr2R:16192019-16196170 (-) // 100.0 //	1.1747	down	0.0451
1633506_s_at	*CG6091*	chr3L:11599121-11602540 (-) // 100.0 //	1.1026	up	0.0241
1627394_s_at	*aop*	chr2L:2156483-2178749 (-) // 100.0 //	1.1305	up	0.0161
1637461_at	*CG9527*	chr2L:6445069-6448016 (-) // 100.0 //	1.3763	down	0.0240
1637357_at	*CG9463*	chr2L:8765358-8768708 (-) // 100.0 //	2.3100	up	0.0391
1630983_s_at	*CG32736 /// CG42308*	chrX:6905941-6906127 (+) // 100.0 //	1.2269	down	0.0319
1639338_at	*CG11912 /// DsimCG11912*	chr2L:318356-319254 (-) // 100.0 //	1.9346	down	0.0284

1640350_at	*fau*	chr3R:6593938-6603083 (-) // 100.0 //	1.3477	down	0.0383
1638140_at	*CG14043*	chr2L:4971703-4973687 (+) // 100.0 //	1.1558	down	0.0110
1625897_s_at	*tal-1A /// tal-2A /// tal-3A /// tal-AA*	chr3R:9638840-9640372 (+) // 100.0 //	1.2980	down	0.0375
1624943_at	*CG1636*	chrX:8167482-8169450 (+) // 100.0 //	1.1027	down	0.0469
1625276_a_at	*Eip71CD*	chr3L:15504152-15506302 (+) // 100.0 //	1.2323	down	0.0164
1625027_a_at	*GXIVsPLA2*	chr3L:15948274-15949355 (+) // 100.0 //	1.1285	up	0.0272
1634582_at	*CG32736 /// CG42308*	chrX:6905526-6906169 (+) // 100.0 //	1.2337	down	0.0004
1623182_at	*mRpL51*	chr2L:8516753-8517377 (-) // 100.0 //	1.1543	down	0.0497
1627254_at	*CG7427*	chr3L:15556537-15558662 (+) // 100.0 //	1.2492	down	0.0168
1628155_at	*klar*	chr3L:434257-540589 (-) // 100.0 //	1.1552	down	0.0393
1638217_at	*CG10166*	chr2L:19525473-19526324 (-) // 100.0 //	1.1834	down	0.0262
1632277_a_at	*HDAC6*	chrX:15226987-15234388 (+) // 100.0 //	1.1578	up	0.0425
1633914_at	*Gal*	chr2L:6003573-6006791 (-) // 100.0 //	1.6466	up	0.0308
1635634_at	*CG7172*	chr3L:21561234-2156198	1.2645	down	0.0415

		8 (-) // 100.0 //			
1628281_at	*Mo25*	chr3L:16606317-16608572 (-) // 100.0 //	1.1307	down	0.0474
1640024_at	*CG7598*	chr3R:25562691-25563792 (+) // 100.0 //	1.0993	down	0.0289
1628705_at	*PH4alphaEFB*	chr3R:26291773-26311521 (+) // 100.0 //	1.6226	down	0.0032
1627216_s_at	*Zyx102EF*	chr4:1077990-1081542 (-) // 99.55 //	1.2127	up	0.0027
1626439_at	*CG15353*	chr2L:2006753-2007193 (-) // 100.0 //	1.5174	down	0.0182
1627823_at	*mRpL35*	chr3R:17849265-17850069 (+) // 100.0 //	1.2512	down	0.0044
1629308_at	*CG17192*	chr3R:22842934-22844004 (-) // 100.0 //	2.0784	down	0.0193
1630207_at	*CG16972*	chr2L:13197091-13203269 (-) // 100.0 //	1.1104	up	0.0307
1637814_s_at	*Jafrac2*	chr3L:3042374-3044181 (+) // 100.0 //	1.0688	up	0.0077
1636815_a_at	*numb*	chr2L:9437512-9463293 (+) // 100.0 //	1.1119	down	0.0320
1640512_at	*nmdyn-D7*	chr3R:5505662-5507224 (+) // 100.0 //	1.1194	up	0.0131
1623813_at	*CG33307*	chr2L:13840801-13843742 (-) // 100.0 //	2.0993	up	0.0175
1631639_at	*TpnC47D*	chr2R:7161537-7162796 (-) // 100.0 //	1.5684	down	0.0095

1625392_at	*mRpL42*	chr2R:5954153-5954748 (+) // 100.0 //	1.1326	down	0.0292
1639256_at	*CG5676*	chr2L:10254603-10255884 (-) // 100.0 //	1.6027	up	0.0265
1640415_at	*CG3860*	chr2R:19879660-19882573 (-) // 100.0 //	1.0928	down	0.0276
1628482_at	*Rab39*	chrX:7628958-7630510 (+) // 100.0 //	1.0967	up	0.0479
1640659_s_at	*CG6664*	chr3L:17006173-17009280 (-) // 100.0 //	1.0826	down	0.0474
1626456_at	*l(1)G0320*	chrX:9445637-9447703 (-) // 100.0 //	1.2226	down	0.0294
1629969_at	*CG11426*	chr3L:22463657-22466610 (+) // 100.0 //	2.5528	up	0.0410
1637394_s_at	*CG31781*	chr2L:16869319-16870181 (+) // 100.0 //	1.5469	up	0.0478
1633779_s_at	*loj*	chr3L:6088092-6089615 (+) // 100.0 //	1.3303	down	0.0402
1622946_at	*CG6908*	chr3R:7514098-7515745 (-) // 100.0 //	1.1972	down	0.0063
1637072_at	*CG11459 /// DsimCG11459*	chr3R:1851831-1852962 (-) // 100.0 //	6.3044	up	0.0368
1624310_s_at	*CG4753*	chr3L:16372069-16374670 (-) // 100.0 //	1.2516	down	0.0142
1629398_at	*CG10383*	chr2L:18690901-18695422 (-) // 100.0 //	1.4105	up	0.0240
1628629_at		chr2R:7783495-7784193 (-) // 100.0 //	1.1325	up	0.0308

1636943_s_at	*Spn5*	chr3R:11028782-11032147 (-) // 100.0 //	1.0528	up	0.0358
1626458_at	*Sirt2*	chr3R:16154191-16155753 (+) // 100.0 //	1.1226	up	0.0416
1628472_at	*deltaCOP*	chrX:1753175-1755825 (-) // 100.0 //	1.0963	down	0.0254
1625014_at	*Ilk*	chr3L:21209993-21212347 (+) // 100.0 //	1.1089	up	0.0249
1625692_s_at	*CG42379 /// CG42380 /// CG42381 /// CG9865*	chr2R:17555031-17557560 (-) // 100.0 //	1.0600	down	0.0138
1636999_a_at	*MESK2*	chr2R:17389590-17401091 (+) // 99.39 //	1.0992	up	0.0317
1635678_at	*CG11299*	chr2R:19601443-19620709 (+) // 100.0 //	1.2013	up	0.0209
1623436_a_at	*bun*	chr2L:12457130-12461237 (-) // 100.0 //	1.2747	up	0.0189
1630981_at	*CG2698*	chr3R:3827305-3831432 (+) // 100.0 //	1.0667	up	0.0140
1623194_at	*CG7637*	chr2R:6698060-6698395 (-) // 98.54 //	1.3563	down	0.0317
1631240_at	*OstStt3*	chr3R:20892433-20895372 (-) // 100.0 //	1.1710	down	0.0377
1623969_at	*CG31200*	chr3R:16569955-16571060 (-) // 100.0 //	1.6494	up	0.0425
1628290_s_at	*CG17292*	chr2L:8370512-8374250 (+) // 99.51 //	1.2006	up	0.0009

1631931_s_at	*Sdc*	chr2R:17281121-17368673 (-) // 100.0 //	1.1960	up	0.0245
1622950_a_at	*CG10069*	chr2R:17169804-17173174 (+) // 100.0 //	1.1906	up	0.0317
1635740_at	*Hmu*	chr3R:23114492-23117308 (+) // 99.83 //	1.1306	down	0.0418
1634972_at	*CG4802*	chr2R:13315224-13316816 (+) // 100.0 //	1.1526	down	0.0347
1626367_at	*Sgt*	chr2L:17473482-17474773 (+) // 100.0 //	1.1019	down	0.0250
1639410_at	*ARP-like*	chr3R:12175134-12176526 (+) // 100.0 //	1.1766	down	0.0145
1636920_s_at	*CG12016*	chr3L:3319556-3322491 (+) // 100.0 //	1.1777	up	0.0101
1638120_at	*CG1927*	chr3L:1966722-1970075 (-) // 100.0 //	1.1926	up	0.0027
1641190_at	*Jon65Aii*	chr3L:6045449-6046291 (+) // 100.0 //	1.4153	down	0.0069
1636639_at	*Spp*	chr2L:542775-544627 (-) // 100.0 //	1.2757	down	0.0307
1632259_at		chr3R:7798807-7800587 (-) // 100.0 //	1.1050	up	0.0227
1634844_at	*CG9577*	chrX:20060707-20062119 (+) // 100.0 //	1.1000	down	0.0276
1638812_at	*CG11501*	chr3R:25319573-25319987 (+) // 100.0 //	21.3035	up	0.0249
1625824_at	*CG9779 /// DsimCG9779*	chr3R:135363-136667 (-) // 100.0 //	1.1138	up	0.0491

1634992_s_at	*CG9821 /// DsimCG9821*	chr3R:4643368-4646386 (-) // 99.7 //	1.1116	up	0.0403
1629082_at	*CG14407*	chrX:14731499-14732230 (-) // 100.0 //	1.0685	down	0.0444
1628991_at	*colt*	chr2L:2768617-2770066 (-) // 100.0 //	1.0830	down	0.0396
1640523_at	*mRpL28*	chr2L:4973977-4975283 (+) // 100.0 //	1.1155	down	0.0068
1632481_at	*CG8602*	chr3L:7319372-7321958 (-) // 100.0 //	1.1761	up	0.0498
1631028_s_at	*l(2)k09913*	chr2R:18954724-18959398 (+) // 100.0 //	1.1525	down	0.0172
1626304_at	*CG32446*	chr3L:21634141-21635699 (+) // 100.0 //	1.1617	down	0.0312
1641295_s_at	*KdelR*	chr2L:10426866-10428159 (+) // 100.0 //	1.1025	down	0.0395
1637678_at	*CG10340*	chr3R:12439133-12440109 (-) // 100.0 //	1.1432	down	0.0015
1634773_at	*SsRbeta*	chr3L:16122739-16124086 (+) // 100.0 //	1.1700	down	0.0365
1637701_at	*Cpr49Ab*	chr2R:8265961-8266846 (+) // 100.0 //	1.5768	down	0.0313
1641454_at	*CG1461*	chrX:14155255-14158879 (+) // 100.0 //	1.3365	up	0.0420
1633401_s_at	*Cyp12d1-d /// Cyp12d1-p*	chrUextra:247021-248794 (+) // 99.81 // /// chr2R:7007609-7009382 (-) // 100.0 // ///	1.0895	down	0.0433

		chr2R:7011375-7013148 (-) // 99.81 //			
1627845_at	*CG6673*	chr3L:8515512-8516600 (-) // 100.0 //	1.0906	up	0.0330
1625847_at	*CG9034*	chrX:9053544-9054018 (-) // 99.05 //	1.1911	down	0.0299
1637089_at	*Syb*	chr2R:6136981-6139914 (-) // 100.0 //	1.1063	up	0.0395
1625223_a_at	*stck*	chr3R:4182688-4185745 (+) // 100.0 //	1.1754	up	0.0160
1631227_at	*CG5793*	chr3R:16953445-16954630 (+) // 100.0 //	1.2266	up	0.0271
1630929_at	*CG5885*	chr2L:9917220-9918165 (-) // 100.0 //	1.2290	down	0.0213
1625164_at	*TotX*	chr3R:16730637-16731240 (-) // 100.0 //	7.7451	up	0.0431
1637444_s_at	*pug*	chr3R:6522239-6529011 (+) // 100.0 //	1.4267	up	0.0276
1640666_at	*CG31205*	chr3R:16556141-16558264 (+) // 100.0 //	2.5848	up	0.0230
1623788_at	*CG7461*	chr2R:15192644-15194689 (+) // 100.0 //	1.2039	down	0.0101
1638663_at	*Sec61alpha*	chr2L:6477532-6480489 (-) // 100.0 //	1.2231	down	0.0045
1629433_at	*CG12091*	chr3L:1535011-1536831 (-) // 100.0 //	1.1560	up	0.0409
1635450_a_at	*smp-30*	chr3R:10571811-10573043 (-) // 100.0 //	1.5009	up	0.0127

1624488_a_at	*CG17734*	chr3R:7067921-7069698 (-) // 100.0 //	1.1715	down	0.0489
1639441_at	*Crc*	chr3R:5454656-5456607 (-) // 100.0 //	1.0844	down	0.0181
1638361_at	*CG12374*	chr2R:8649616-8651330 (+) // 100.0 //	2.0328	down	0.0090
1627382_at	*Sec61beta*	chr2R:10506231-10507189 (+) // 100.0 //	1.1190	down	0.0055
1626566_at	*CG12262*	chr3L:7712460-7714585 (+) // 100.0 //	1.0656	down	0.0346
1624715_at	*mtacp1*	chr3L:1329805-1331633 (+) // 100.0 //	1.1556	down	0.0248
1622947_s_at	*porin*	chr2L:10847226-10850868 (-) // 100.0 //	1.1068	down	0.0067
1634918_a_at	*CG32276*	chr3L:3197859-3199043 (-) // 100.0 //	1.1095	down	0.0164
1640599_at	*ade5*	chrX:12651774-12654651 (-) // 100.0 //	1.2425	up	0.0132
1639892_at	*Sodh-1*	chr3R:2878056-2880264 (+) // 100.0 //	1.2521	up	0.0169
1635548_s_at	*CG30287 /// CG34166*	chr2L:14743252-14743860 (+) // 100.0 //	1.2659	up	0.0183
1639323_at	*TotC*	chr3R:16698709-16699310 (+) // 100.0 //	9.2596	up	0.0489
1632667_s_at	*Gapdh2*	chrX:15762335-15763662 (+) // 100.0 //	1.0259	down	0.0315
1623635_at	*TotM*	chr2L:5329856-5330466 (+) // 100.0 //	15.3075	up	0.0082

1635549_at	*TotA*	chr3R:16696757-16697427 (+) // 100.0 //	10.5306	up	0.0270

Supplementary Table S2. The Primers specific to each of the 10 genes for RT-PCR

Gene symbol	Primer direction	Sequence	Temperature (℃)
Numb	Forward Primer	CTGTGCTTTTGCCGTTTGTCT	60.5
	Reverse Primer	TACGCTCCGCTCATTTGTGC	62.1
Tom	Forward Primer	CAGGGTCAGCAACAGAGCATC	60.4
	Reverse Primer	GAACCCAACGGTCGAACTGC	62.2
Osa	Forward Primer	CCGCTATCCGCCTCAACAAG	63.1
	Reverse Primer	TGAGCCCGGAGGTGCTATTC	62.4
Nedd4	Forward Primer	TGACACGCGACGACTTCCTG	62.6
	Reverse Primer	GGCGCAGAGTGTAGCTTTGTTC	61.8
Bun	Forward Primer	GGTGGCACTGGCGGTAACAT	63.1
	Reverse Primer	CGCAAACTTTCGCTGGTGGT	62.7
Sca	Forward Primer	CGATCAGGTGCGCCTGTTG	63.0
	Reverse Primer	CGCTGTTCGCATTCGTGGTG	65.0
Stck	Forward Primer	ACGAGGAGCCTTTGCGATTC	60.7
	Reverse Primer	CTCTTGACCTCCCGAGCAGT	61.2
Ilk	Forward Primer	GCGACGACATCCCACTCCAT	62.7
	Reverse Primer	TGCTCGTTTACCGCATTCAC	62.3
Hth	Forward Primer	TTTCAGCATTTAACGCATCCC	60.5
	Reverse Primer	TTATTCACTTGCAGTATCGTTAGGC	60.4
Dad	Forward Primer	AAGGTGTTATGGCGTTATGCG	60.7
	Reverse Primer	ATGGCTGTTGTTGTTGTTGTGG	60.5
Gapdh	Forward Primer	GCGTCATCGACCTGATCAAGT	62.0
	Reverse Primer	CCTTGCGGATTATGCAACAGT	61.8

Supplementary Table S3. The fold change and transcript abundance identified using DNA microarray and qRT-PCR analyses in *Drosophila melanogaster* with wing abnormalities induced by high hydrostatic pressure

Gene Symbol	Location	Function	Process	Transcript levels using microarray	Transcript levels using RT-PCR	Concordant
Numb	Chromosome 2L, NT_033779.4 9437469..9464184	Protein binding	Notch signaling pathway	-1. 1	+2.9	-
Tom	Chromosome 3L, NT_037436.3 14962444..14963406	Protein binding	Notch signaling pathway	-16.9	-2.1	+
Sca	Chromosome 2R, NT_033778.3 8668049..8689515	Signal transducer activity	Imaginal disc-derived wing margin morphogenesis	-1.7	-2.0	+
Bun	Chromosome 2L, NT_033779.4 12455540..12546611	Transcription factor activity	Notch signaling pathway	+1.3	+1.1	+
Nedd4	Chromosome 3L, NT_037436.3 17523181..17539760	Notch binding	Notch signaling pathway	+1.2	+1.0	+

Stck	Chromosome 3R, NT_033777.2 4182689..4185686	Protein binding	Apposition of dorsal and ventral imaginal disc-derived wing surfaces	+1.2	+1.1	+
Ilk	Chromosome 3L, NT_037436.3 21210001..21212340	Protein kinase activity	Apposition of dorsal and ventral imaginal disc-derived wing surfaces	+1.1	+2.1	+
Osa	Chromosome 3R, NT_033777.2 13513540..13544072	Transcription co-activator activity	Imaginal disc-derived wing margin morphogenesis	+1.2	+3.1	+
Hth	Chromosome 3R, NT_033777.2 6333283..6465290	Transcription factor activity	Imaginal disc-derived wing morphogenesis	+1.5	+1.9	+
Dad	Chromosome 3R, NT_033777.2 12879729..12896135	Transcription factor activity	Imaginal disc-derived wing morphogenesis	+1.4	+2.3	+
